# Three-Dimensional Strain for Left Ventricular Function Assessment in Children After Successful Ventricular Septal Defect Repair

**DOI:** 10.3390/jcm15208199

**Published:** 2026-10-21

**Authors:** Halszka Kamińska, Julia Haponiuk-Skwarlińska, Radosław Pietrzak, Bożena Werner

**Affiliations:** 1 Department of Pediatric Cardiology and General Pediatrics, Medical University of Warsaw, 61 Żwirki i Wigury Street, 02-091 Warsaw, Poland; radoslaw.pietrzak@wum.edu.pl (R.P.); bozena.werner@wum.edu.pl (B.W.); 2 Department of Pediatric Cardiology and General Pediatrics, Doctoral School, Medical University of Warsaw, 61 Żwirki i Wigury Street, 02-091 Warsaw, Poland; julia.haponiuk-skwarlinska@wum.edu.pl

**Keywords:** AI, children, echocardiography, three-dimensional strain, ventricular septal defect

## Abstract

**Background:** Ventricular septal defect (VSD) has a good prognosis after surgical treatment. Three-dimensional (3D) strain echocardiography is a novel method adapting AI for assessing ventricular function; however, its utility remains to be tested. The aim of the study was to assess accuracy of 3D strain echocardiography based on a specific population of patients. **Methods:** The study enrolled children after successful surgical VSD treatment. In all patients, 3D echocardiography was performed to assess left ventricular ejection fraction (LVEF) and global and segmental longitudinal (LS), circumferential (CS), and radial strain (RS) values. The results were compared with those of healthy individuals matched for age and sex. **Results:** The study group consisted of 20 children (12 males) aged 3–17 years (mean 7.0 ± 4.3 years), with normal ventricular function on routine 2D echocardiography. The global strain values in all patients were within the standard range in the literature for sex and age. Statistically significant differences between study and control groups were observed in terms of LVEF (55.6 ± 6.9% vs. 59.3 ± 4.8%, *p* = 0.046), global LS (−21.4 ± 4.3 vs. −24.8 ± 3.5, *p* = 0.010), and RS (38.7 ± 6.1% vs. 43.6 ± 5.0%, *p* = 0.010). Segmental strain values showed dysfunction limited to the patched septal area. **Conclusions:** 3D strain echocardiography is able to detect regional subclinical cardiac function impairment in children after successful surgical treatment of VSD.

## 1. Introduction

Ventricular septal defect (VSD) is the most common congenital heart defect in children, with positive outcomes and good postoperative prognosis in cases requiring surgical treatment [1,2]. Children after successful repair are considered healthy and do not commonly require lifestyle restrictions, including full participation in normal physical activity. In routine follow-up two-dimensional echocardiography generally shows normal ventricular ejection fraction [3]. Nevertheless, surgical treatment is consistent with suturing an akinetic patch on the ventricular septum; therefore, regional systolic dysfunction is bound to be present in the area of the cardiac muscle [4].

Three-dimensional (3D) strain echocardiography is known as a novel, fast and sensitive method for automatic ventricular function assessment, offering longitudinal, circumferential and radial strain values, both global and segmental, based on 1–2 heartbeats of 3D acquisition. We know that strain echocardiography is the most sensitive tool for detecting even subclinical systolic left ventricular dysfunction, preceding actual ejection fraction deterioration; however utility of this method’s 3D version in pediatric cardiology is still to be tested and trained on constantly evolving AI-based algorithms to make it more accessible and replicable for users with different levels of experience [5,6,7,8,9,10,11].

The patients who were successfully surgically treated for VSD were selected for this study to test the accuracy, sensitivity, and potential benefits of the method, as we were aware of where exactly to expect lower muscle contractility.

## 2. Materials and Methods

The exploratory retrospective–prospective observational study enrolled consecutive children scheduled for routine follow-up after successful surgical VSD treatment, with no residual shunting, no coexisting heart problems and with standard echocardiograms within normal limits. The enrollment process was conducted from January to December 2025 in the Department of Pediatric Cardiology and General Pediatrics, Medical University of Warsaw in Poland and included all patients who met the abovementioned criteria. The enrollment process was conducted from January to December 2025—with around 25% of patients followed up until 30.06.2025 (retrospective) or later on—as a prospective study. Each patient was assessed only once at a distinct time period after surgery.

To provide a reference for the echocardiographic assessment, a case–control group of 20 healthy children matched by sex and age was included (Table 1). The group consisted of children referred for echocardiography (i.e., for routine screening, qualification for sports classes, and diagnostic workup of chest pain, with an innocent heart murmur) and those with heart disease, significant systemic disease, congenital heart disease, or other conditions expected to affect cardiac structure or function were excluded. In all children, the echocardiographic results were within normal limits. Patients were recruited both prospectively (70%) and from an already existing registry for the best possible match.

All patients underwent echocardiography without sedation performed by an experienced (>15 years of practice) pediatric cardiologist with 3D data acquisition on an EPIQ CVx 5.0 ultrasound machine (Philips Medical System, WA, USA; software version 9.0.16) using the X5-1 transducer (single apical take from 2 heartbeats with the frame rate > 20 Hz; mean time of acquisition under 1 min); it was analyzed further offline using 4D LV-Analysis software (Philips Medical System, WA, USA; Tomtec software version 6.0), automatically assessing the left ventricular ejection fraction (LVEF), global longitudinal strain (LV-GLS), global circumferential strain (LV-GCS) and global radial strain (LV-GRS) with further analysis of segmental strain values; the manual corrections of the endocardial contouring were generally avoided and applied only when necessary (in under 10% of cases) for the best possible evaluation of the algorithm. The offline analysis was performed by two investigators with different levels of experience who were blinded to patients’ status (study or control group) using the fellow researcher’s results for unbiased assessment of interobserver reliability; the process of analysis for a single patient lasted less than 2 min.

The study was approved by the University Ethics Committee and was performed in accordance with the ethical standards laid down in the 1964 Declaration of Helsinki and its later amendments.

Statistical analysis was performed using IBM SPSS version 30.0.0. Medians, proportions, and percentages were used to describe the population and resulting observations. The Shapiro–Wilk test was used to assess whether the values of quantitative variables came from a normally distributed population. The comparisons between the groups were performed with a T-test for normally distributed data and a Mann–Whitney U test for non-normally distributed data. For categorical variables Fisher’s exact test was used. The significance level was set at α = 0.05 for all applied tests.

Missing data were assessed for each variable prior to statistical analysis. Individuals with missing values were excluded from the analysis.

Potential outliers were assessed based on the distribution of individual variables and were not excluded solely on the basis of their statistical extremeness.

The interobserver reliability analysis was performed using the Bland–Altman method.

## 3. Results

The study group consisted of 20 children (12 males) aged 3–17 years (mean 7.0 ± 4.3 years) in whom cardiac surgery was performed during the first year of life (2.5–16.2, mean 7.9 years after treatment), with no symptoms of heart failure or arrhythmia and with normal ventricular function in routine 2D echocardiography.

The 3D data set registration proved to be time-efficient (mean of 30 s per patient), with additional time spent on offline analysis comprising less than 2 min. No sedation was used.

The strain values in all patients were within the normal range in the literature for sex and age.

**Figure 1:**
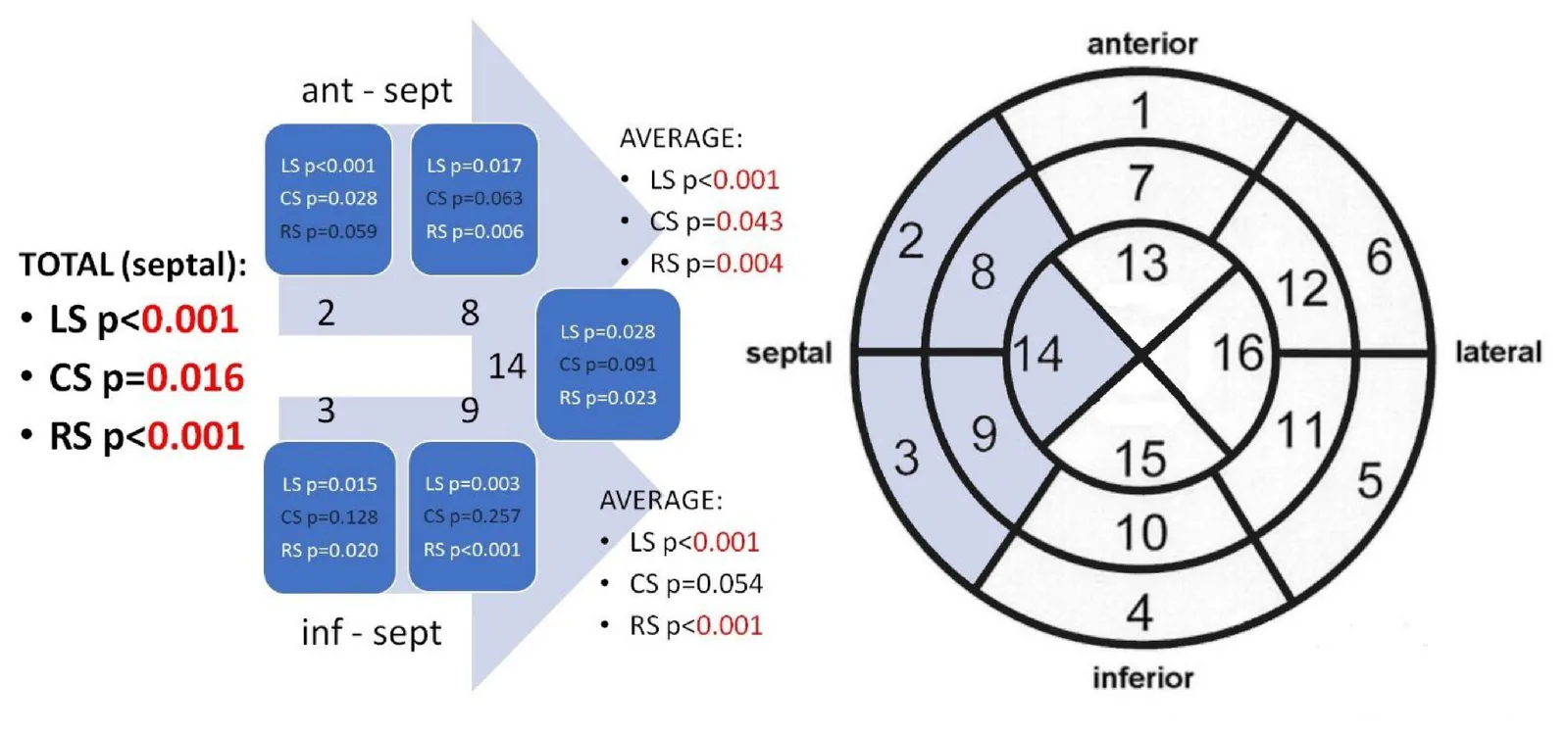
Graphical presentation of comparison between study and control groups for single segments of the interventricular septum. LS: longitudinal strain; CS: circumferential strain; RS: radial strain. Segmental strain analysis of the left ventricle; comparison between study and control groups. Statistically significant differences between the study and control groups are marked in blue.

Statistically significant differences between the study and control groups were observed globally for LVEF (55.6 ± 6.9% vs. 59.3 ± 4.8%, *p* = 0.046) and in 3D strain values for LV-GLS and LV-GRS. The differences in terms of LV-GCS were not statistically significant. The segmental strain analysis of the singled-out septal area of the ventricle is presented in Figure 1.

The results of global and segmental strain analysis of the ventricle are presented in Table 2. The means of segmental longitudinal, circumferential, and radial strain values for the whole septum were significantly lower in comparison with healthy children. In single septal segments, the differences were generally marked for longitudinal and radial strain values, with no significant differences in circumferential strain.

The lack of statistically significant differences was noted between corresponding segments outside the septal area in the study and control groups (with the exception of segment 10).

The high interobserver reliability is illustrated in Table 3.

## 4. Discussion

In our studied population of children after successful surgical VSD treatment, the mean values of 3D global and segmental strain were within normal limits according to the published literature [5,7,8,9,12]. This tendency aligns with common knowledge of a universally good postoperative prognosis in this group of patients, as illustrated by many studies [1,3,13,14,15]; however, the majority only report global functional data, without single-segment analysis.

We chose to study this specific population of patients in an attempt to test the sensitivity and accuracy of 3D strain echocardiography, knowing that our patients are bound to present some, even if subclinical, dysfunction in the patched septal area of the heart muscle.

The wide span of age in our studied population was mirrored in the control group, providing a chance to minimize the age-dependency of 3D strain values, which is still not well documented in the literature [6,8,11].

The comparison between the study and control groups in our study showed a discrepancy (with significantly lower absolute values) in global longitudinal and radial strain, whereas global circumferential strain was not significantly different. This is an interesting contrast to the data published by Ali et al. [1], where global circumferential strain was the only parameter significantly impaired in long-term follow-up of patients after interventional VSD closure in comparison with healthy individuals, and no correspondence was found between increased left ventricular volumes and the potential to generate higher CS mechanically according to the Frank–Starling law.

All our studied patients received an early operation (in the first year of life), with a mean time of 7.9 years after surgery and a marked span in the time to follow-up of control points. The similar cohort analyzed by Nederend et al. [3] showed normal left ventricular function, even in cases with lower exercise tolerance, over 10 years after surgery. Veeram Reddy et al. [4] studied left ventricular dyssynchrony in 3D echocardiography in children more than 5 years after VSD treatment; however, abnormalities were apparent only in patients with right bundle branch block (RBBB).

In our results, segmental 3D strain analysis of the whole left ventricular muscle showed marked discrepancies in comparison with healthy individuals, limited to the surgically patched septal area, advocating for the high accuracy of the method.

The importance of this finding may be disputable in our studied population, as children after successful VSD treatment with normal global left ventricular function are still considered unaffected by segmentally reduced strain in the patched area. Further potential directions to study include the long-term follow-up of patients with longitudinal assessment of 3D strain results and their relation to possible evolution of remote complications, such as arrhythmias or clinical cardiac systolic or diastolic dysfunction. Accepting that the abovementioned side effects are rarely observed in pediatric populations with good results of surgical treatment, it is worth noting and following subtle and still subclinical myocardial dysfunctions even in an asymptomatic cohort traditionally considered to experience excellent surgical outcomes.

Furthermore, the demonstrated precision of the 3D strain may be useful in the follow-up of global and regional ventricular function in a population of patients requiring meticulous control of contractility. Specifically, children with cardiomyopathies and progressing heart failure, during pharmacotherapy or after cardiotoxic treatment, may benefit from sensitive functional assessment to improve their quality of control and long-term management.

Additionally, the high accuracy and interobserver reliability of the automatic method of 3D strain analysis may be employed in many clinical scenarios with minimal additional time investment.

## 5. Limitations

Our study is exploratory in character and the sample size is small. The patients were derived from a single center, with a wide age range indicating that they varied in the span of the follow-up stage, and were assessed at a single time-point with no longitudinal follow-up data.

All participants (from both the study and control group) were examined with the same vendor equipment and analyzed with the same software.

In our assessment multiple comparisons were used without statistical correction; therefore, *p*-values close to the significance threshold should be interpreted with caution and in the context of the overall pattern of findings rather than as isolated evidence.

## 6. Conclusions

3D strain echocardiography is able to detect regional subclinical cardiac function impairments in children after successful surgical treatment of VSD.

## Figures and Tables

**Table 1:** Baseline characteristics in study and control groups.

	Study Group *n* = 20	Control Group *n* = 20	*p* Value
Age (years)	7.0 ± 4.3	7.7 ± 3.58	0.62
Sex	12 males, 8 females	13 males, 7 females	1.0
Height (cm)	120.2 ± 27.8	126.5 ± 21.8	0.44
Weight (kg)	25.7 ± 14.5	29.8 ± 13.7	0.38
BSA (m^2^)	0.91 ± 0.36	1.01 ± 0.32	0.36

**Table 2:** Segmental strain analysis of the left ventricle; comparison between study and control group.

Parameter	Study Group (*n* = 20)	Control Group (*n* = 20)	*p*-Value	Effect Size	95% CI
**Longitudinal strain**					
**Average (GLS)**	−21.4 ± 4.3	−24.8 ± 3.5	0.010 ^t^*	0.853	0.200–1.497
**1 [ant]**	−24.2 (−25.6; −20.5)	−24.0 (−29.2; −20.4)	0.820 ^u^	0.378	−0.259–1
**2 [ant/sept]**	−18.3 (−20.7; −14.2)	−23.5 (−26.7; −21.0)	<0.001 ^u^*	1.159	0.480–1.824
**3 [inf/sept]**	−20.2 (−22.2; −14.2)	−23.8 (−26.2; −21.6)	0.015 ^u^*	0.627	−0.012–1.234
**4 [inf]**	−24.1 ± 5.4	−25.9 ± 4.8	0.261 ^t^	0.360	−0.267–0.983
**5 [inf/lat]**	−25.4 (−30.1; −22.6)	−27.7 (−30.5; −22.5)	0.799 ^u^	−0.080	−0.699–0.541
**6 [ant/lat]**	−26.1 (−31.8; −22.9)	−25.8 (−32.4; −21.2)	0.883 ^u^	−0.20	−0.649–0.600
**7 [ant]**	−22.1 ± 7.2	−24.5 ± 6.8	0.283 ^t^	0.344	−0.283–0.967
**8 [ant/sept]**	−17.1 ± 5.4	−22.0 ± 7.0	0.017 ^t^*	0.791	0.142–1.431
**9 [inf/sept]**	−18.5 ± 7.0	−24.3 ± 4.2	0.003 ^t^*	1.00	0.343–1.662
**10 [inf]**	−20.0 ± 7.0	−23.8 ± 2.8	0.034 ^t^*	0.709	0.65–1.344
**11 [inf/lat]**	−21.1 ± 7.0	−23.8 ± 3.4	0.128 ^t^	0.496	0.137–1.123
**12 [ant/lat]**	−20.8(−28.1; −18.4)	−23.3 (−28.9; −19.7)	0.231 ^u^	0.288	−0.337–0.909
**13 [ant]**	−21.0 ± 6.8	−23.0 ± 7.3	0.367 ^t^	0.289	−0.336–0.910
**14 [sept]**	−19.1 (−24.4; −16.1)	−23.2 (−27.6; −18.0)	0.023 ^u^*	0.639	−0.001–1.271
**15 [inf]**	−22.4 ± 7.0	−24.7 ± 3.5	0.196 ^t^	0.417	−0.213–1.041
**16 [lat]**	−23.1 ± 7.0	−25.0 ± 5.8	0.356 ^t^	0.296	−0.330–0.917
**Circumferential strain**					
**Average (GCS)**	−24.2 ± 5.2	−26.6 ± 4.7	0.129 ^t^	0.491	−0.142–1.117
**1 [ant]**	−25.6 ± 10.3	−27.0 ± 6.5	0.594 ^t^	0.170	−0.452–0.790
**2 [ant/sept]**	−20.1 ± 10.8	−25.9 ± 7.6	0.057 ^t^*	0.623	−0.16–1.255
**3 [inf/sept]**	−19.9 ± 8.9	−23.1 ± 8.6	0.257 ^t^	0.364	−0.264–0.987
**4 [inf]**	−17.9 ± 13.3	−22.0 ± 11.0	0.290 ^t^	0.340	−0.287–0.962
**5 [inf/lat]**	−21.1 ± 10.6	−19.7 ± 8.0	0.637 ^t^	−0.150	−0.770–0.471
**6 [ant/lat]**	−20.9 ± 10.5	−17.6 ± 6.4	0.246 ^t^	−0.374	−0.997–0.254
**7 [ant]**	−29.1 (−35.1, −21.3)	−26.4 (−32.5; −24.8)	0.779 ^u^	0.111	−0.510–0.731
**8 [ant/sept]**	−24.1 ± 11.4	−30.0 ± 8.0	0.064 ^t^	0.606	−0.032–1.237
**9 [inf/sept]**	−27.0 ± 9.3	−34.0 ± 8.5	0.016 ^t^*	0.796	0.146–1.436
**10 [inf]**	−23.4 ± 11.7	−31.4 ± 10.7	0.033 ^t^*	0.702	0.058–1.337
**11 [inf/lat]**	−28.3 ± 8.6	−30.1 ± 11.0	0.559 ^t^	0.187	−0.436–0.807
**12 [ant/lat]**	−32.1 ± 7.8	−29.2 ± 6.3	0.210 ^t^	−0.404	−1.028–0.225
**13 [ant]**	−23.1 ± 8.2	−23.5 ± 7.3	0.884 ^t^	0.047	−0.574–0.666
**14 [sept]**	−21.9 ± 8.5	−26.4 ± 7.8	0.091 ^t^	0.548	−0.087–1.177
**15 [inf]**	−25.0 ± 6.5	−28.7 ± 8.8	0.143 ^t^	0.474	−0.158–1.100
**16 [lat]**	−30.7 ± 8.2	−25.3 ± 8.4	0.043 ^t^*	−0.661	−1.294–(−)0.020
**Radial strain**					
**Average (GRS)**	38.7 ± 6.1%	43.6 ± 5.0%	*0.010*^t^*	*−0.893*	*−1.539–(−)0.237*
**1 [ant]**	40.8 ± 12.2	44.3 ± 9.4	0.307 ^t^	−0.328	−0.950–0.299
**2 [ant/sept]**	31.3 ± 9.8	41.6 ± 7.6	*<0.001*^t^*	*−1.171*	*−1.838–(−)0.492*
**3 [inf/sept]**	34.6 ± 8.8	40.1 ± 7.0	*0.037*^t^*	*−0.685*	*−1.319–(−)0.042*
**4 [inf]**	36.0 ± 13.6	41.0 ± 9.5	0.190 ^t^	−0.432	−1.048–0.206
**5 [inf/lat]**	40.3 ± 10.6	38.6 ± 8.7	0.602 ^t^	0.166	−0.456–0.786
**6 [ant/lat]**	39.7 (29.2; 52.8)	40.5 (30.8; 44.8)	0.640 ^u^	−0.020	−0.640–0.600
**7 [ant]**	41.4 ± 12.8	44.5 ± 10.8	0.417 ^t^	−0.260	−0.880–0.365
**8 [ant/sept]**	34.6 ± 10.9	43.9 ± 9.0	*0.006*^t^*	*−0.926*	*−1.574–(−)267*
**9 [inf/sept]**	38.2 ± 9.6	49.1 ± 9.3	*<0.001*^t^*	*−1.291*	*−1.968–(−)0.600*
**10 [inf]**	36.8 ± 11.3	46.5 ± 9.3	*0.005*^t^*	*−0.936*	*−1.585–(−)0.276*
**11 [inf/lat]**	41.6 ± 10.7	45.6 ± 10.4	0.246 ^t^	−0.373	−0.996–0.255
**12 [ant/lat]**	46.5 ± 9.8	45.9 ± 8.0	0.842 ^t^	0.064	−0.557–0.683
**13 [ant]**	37.4 ± 11.2	39.5 ± 10.9	0.545 ^t^	−0.193	−0.813–0.429
**14 [sept]**	35.6 (28.2; 44.1)	41.3 (35.7; 49.1)	*0.023*^u^*	*−0.722*	*−1.358–(−)0.077*
**15 [inf]**	40.1 ± 8.1	45.2 ± 7.5	*0.049*^t^*	*−0.643*	*−1.275–(−)0.002*
**16 [lat]**	45.4 ± 10.7	42.7 ± 9.4	0.410 ^t^	0.264	−0.361–0.884

* represents statistical significance; ^t^ *t*-test; ^u^ Mann–Whitney U test; GLS: global longitudinal strain; GCS: global circumferential strain; GRS: global radial strain; ant: anterior; sept: septal; lat: lateral; inf: inferior.

**Table 3:** Interobserver reliability (Bland–Altman method).

Parameter	Mean Difference (Bias)	95% CI for Bias	SD of Differences	Lower Limit of Agreement	Upper Limit of Agreement
GLS 3D average (%)	−0.10	−0.74 to 0.55	0.77	−1.61	1.42
GCS 3D average (%)	0.11	−0.49 to 0.70	0.71	−1.29	1.51
GRS 3D average (%)	−0.15	−0.69 to 0.40	0.66	−1.43	1.14

## Data Availability

Data obtained in the study will be shared upon request.

## Abbreviations

The following abbreviations are used in this manuscript:

AI	Artificial Intelligence
VSD	Ventricular Septal Defect
ECHO	Echocardiography
LV	Left Ventricle
GLS	Global Longitudinal Strain
GCS	Global Circumferential Strain
GRS	Global Radial Strain
LVEF	Left Ventricular Ejection Fraction
